# Clinical effect of altered lipid emulsion composition containing fish oil in postoperative patients following abdominal surgery: a prospective, randomized, open-label, comparative, multicenter phase 3 clinical study

**DOI:** 10.1186/cc12187

**Published:** 2013-03-19

**Authors:** M Keum, S Hong, H Han, D Yoon, J Seo, I Yun

**Affiliations:** 1 Asan Medical Center, Seoul, South Korea; 2 Seoul National University Bundang Hospital, Seongnam, South Korea; 3 Gangnam Severance Hospital, Seoul, South Korea; 4 Samsung Medical Center, Seoul, South Korea; 5 Konkuk University Medical Center, Seoul, South Korea

## Introduction

Omega-3 fatty acids have been shown to decrease inflammatory responses after trauma and surgery resulting in reduction of morbidity and mortality in postoperative patients. The n-6:n-3 polyunsaturated fatty acid (PUFA) ratio in parenteral nutrition (PN) also influences the immune modulation. The aim of this study was to evaluate the optimal ratio of PUFA in postoperative patients following abdominal surgery.

## Methods

In a prospective, randomized, open-label, comparative, multicenter, phase 3 clinical study, we compared the safety and efficacy of a 2.1:1 ratio of n-6:n-3 fatty acid compared with 2.5:1 in postoperative patients requiring PN. Fifty-four patients were assigned to receive PN with Combiflex Omega peripheral^® ^(CFO, low ratio group, *n = *28) or SMOFKabiven peripheral^® ^(KAB, high ratio group, *n = *26) for 3 days after abdominal surgery. Safety and efficacy were monitored daily with laboratory parameters, vital signs, and adverse events from the operation day (day 0) until the end of the study (day 4).

## Results

Total cholesterol (TC) and low-density lipoprotein-cholesterol (LDL-C) levels were less changed significantly in the low ratio group (3 ± 18 vs. 16 ± 23 mg/dl, *P *= 0.027 for TC, 4 ± 12 vs. 12 ± 18 mg/dl, *P *= 0.026 for LDL-C) compared with the high ratio group in postoperative patients. Other laboratory parameters and adverse events did not show statistically significant differences between the groups. See Table 1.

**Table 1:** Lipid emulsion composition of CFO and KAB

	CFO	KAB
LCT (g/l)	60	60
MCT (g/l)	50	60
Olive oil (g/l)	50	50
Fish oil (g/l)	40	30
n-6:n-3	2.1:1	2.5:1

## Conclusion

CFO containing low n-6:n-3 PUFA ratio was comparable with KAB in safety and efficacy in postoperative patients requiring PN and also had advantages with regard to lipid metabolism aspect.
